# Chicago Medical Society

**Published:** 1883-12

**Authors:** 


### Society Reports.

ARTICLE V.

CHICAGO MEDICAL SOCIETY.

The Chicago Medical Society inaugurated the approaching winter sessions or series of meetings on the evening of the 15th inst., after a vacation of two months, and it is hoped the lively interest will be continued as that evinced by the large attendance of members at this time, and the *jeu d'sprit* present will be productive of many valuable and interesting papers and essays ere the close of the current year.

The President, Dr. D. W. Graham, gave a short address of welcome in the most appropriate manner, and Dr. L. H. Montgomery recorded the minutes.

The first paper was an extensive one, being "The History of Insanity in Chicago," compiled from an analysis of three thousand cases treated at the County Insane Asylum during the past twenty years, by Dr. S. V. Clevenger, special pathologist Cook County Insane Asylum. The following synopsis is hereby appended:

Many things have interposed to baffle his endeavors to present an exact history of the medical progress of this asylum, giving statistical information, etc.; for during the time pricr to the great Chicago fire, which destroyed all the county records, and the paucity of asylum papers dating earlier than 1878, the asylum books were kept in a very careless manner, blotted, smeared and full of errors. At this time the superintendent assuming charge had transferred to new pages all the case histories obtainable from previous records and the fuller details obtained from friends of patients. This mass of information has afforded the material for this article, but in analyzing cases earlier than 1878 many sources of error must be eliminated before they can be made available statistically. In *ante bellum* days there was no county asylum; at that time and during the year 1866 insane persons were treated in the wards of the Poor-House, also subsequently and during the period from October 1, 1867, to January 10, 1871. Some very comical stories are told of this *regime*, from which the writer deduced that the welfare of a patient was insignificant alongside the chances of stealing a dollar or so, in those times. January 1, 1871, the Poor-House had outgrown itself, and the necessity for differentiating insanity from pauperism had become apparent enough to justify the erection of a large brick building capable of accommodating 300 patients. A new superintendent was appointed who started out with true medical *animus* to better the condition of the insane, but the book keeping and records were not satisfcatorily kept. January 1, 1875, a change was made in the appointment of superintendent who served until January 1, 1878, at which time the present incumbent's term began, and who served uninterruptedly to the present time. The writer of the paper had incorporated some sixteen different tables, showing the number of admissions each year (male and female), recoveries, improved, sent to State Hospital, died, unknown, also the duration of residence in asylum of male and female patients, occupation, nativity, maximum per cent. minimum per cent. and mean per cent. of those who recovered, improved each year, etc., and lastly a table showing the psychoses of patients admitted during the fiscal year ending August 31, 1883 (re-admissions included), which to elaborate upon without giving the figures complete might do the author an injustice. Suffice to say that in comparing the figures to some writers in medical journals, there is much unnecessary cavil at the term "recovered," as used by asylum statisticians.

One reason for this probably is that where 100 cannot be obtained in footing up any table requiring it, differently based calculations without all the factors will require additional study and greater chance of error.

The author quoted figures of the California asylum at Napa, showing the increase of insanity in that State, from 1 to every

833 persons in 1860, to 1 in 383 in 1880, and that in this proportion there will be one insane person to two sane in the year 2000 in that State. However, this apparent resulting increase occurs in all new countries. Just so it is with the Chicago insane, the proportion of whom to the population is somewhere in the neighborhood of the California figures of 1880 (1 to 383), as there is a great influx of paupers from Europe, "assisted" by the English government, into Chicago. The statistics of the Kankakee and Elgin asylums, so far as they relate to Chicago insane, should form an integral part of future estimates, in connection with our county asylum, in arriving at an idea of the proportion of insane in our city population, with their death and recovery rates.

Several hypertrophied brains and hearts were exhibited, one heart that of a woman, weighing twenty-four ounces; also, a brain weighing fifty-seven ounces, of a Swedish physician, the "pons" portion being unusually developed. The man had been remarkably intelligent during a portion of his life.

Dr. J. G. Kiernan reported a case of "Insanity from Using Quinine," this being the third one that he has observed during the past two years. The two previous cases were reported two years ago in the *Journal of Nervous and Mental Diseases*, 1881. The case to-night is R. B , æt. 38 ; has a sister epileptic; a maternal grandmother and a maternal aunt died from "rush of blood to the head." The patient resembles the maternal side of the house in appearance and disposition. He has never been able to take even a small quantity of beer, for fear of it affecting his head. Having recently come from a malarious district into Chicago, he was attacked by fever of a quotidian type. Upon the advice of a fellow workman he purchased and took ℈i. of quinine sulphate at a dose. In an hour thereafter he was violent and destructive, smashing furniture purposely. His friends called the doctor at this stage. There was a wild, purposeless violence, but no delusion or hallucination present. He was very incoherent and hilarious. This condition disappeared in two hours, he having meanwhile been given a hypodermic of conine, which controlled his movements. A second dose of the quinine led to exactly the same results, and its astrological influence was, therefore, clear. Since disuse of the quinine there has been no further psychical phenomena. The writer knew of but one other case in literature of transitory fury due to quinine. Such cases as these are likely to become of medico-legal importance. He had, however, heard of three instances in which the use of quinine has been alleged as an excuse for certain escapades seemingly the result of intoxication.

Dr. Clevenger inquired of Dr. K. if there was present any predisposing or exciting causes in all his cases. Answered, all the cases had a hereditary or neurotic taint. He then quoted from Wood, who said in 3,000 cases where quinine was administered the heart's action in three of them became depressed, similar to the action of salicylic acid in some cases.

Dr. R. H. Engert had always found that quinine acted as a tonic to the heart, but recited a case where a man in Mississippi had taken a large dose, became delirious from its effects, and died.

Dr. E. Andrews offered the following resolutions, which were adopted :

That the chair appoint a committee of five, of which the president shall be chairman ; that this committee is hereby instructed to confer with the commissioners of the public library, on the subject of increasing the medical department of that library, and endeavor to agree with the commissioners upon a plan of action, and to report the same to this society for ratification.

That this plan shall, if possible, embrace the following features :

1. That the surplus funds now in the treasury of this society shall be appropriated to the purchase of medical and surgical books and journals for the Public Library.

2. That a certain proportion of the annual dues hereafter shall go for the same object, together with such voluntary donations as the Library Committee hereafter to be appointed may succeed in determining.

3. That the Commissioners of the Public Library shall take good care of the works purchased, and add a suitable sum yearly to be applied to the same objects.

4. That this society will claim no exclusive use of the medical books and journals, but we desire that they shall be placed in the consultation department, and not in the loanable list.

Resulting in the following appointments:

Drs. D. W. Graham, Edmund Andrews, Oscar C. DeWolf, G. C. Paoli, E. Ingals, Committee.

The Society adjourned.

